# Camptothecin resistance is determined by the regulation of topoisomerase I degradation mediated by ubiquitin proteasome pathway

**DOI:** 10.18632/oncotarget.16376

**Published:** 2017-03-18

**Authors:** Koji Ando, Ankur K. Shah, Vibhu Sachdev, Benjamin P. Kleinstiver, Julian Taylor-Parker, Moira M. Welch, Yiheng Hu, Ravi Salgia, Forest M. White, Jeffrey D. Parvin, Al Ozonoff, Lucia E. Rameh, J. Keith Joung, Ajit K. Bharti

**Affiliations:** ^1^ Department of Medicine, Division of Hematology Oncology, Boston University School of Medicine, Boston, MA, USA; ^2^ Molecular Pathology Unit, Massachusetts General Hospital, Charlestown, MA, USA; ^3^ Department of Pathology, Harvard Medical School, Boston, MA, USA; ^4^ Department of Biomedical Informatics, The Ohio State University Comprehensive Cancer Center, Columbus, OH, USA; ^5^ Department of Medical Oncology and Therapeutics Research, City of Hope, Duarte, CA, USA; ^6^ Department of Biological Engineering, Koch Institute for Integrative Cancer Research, Massachusetts Institute of Technology, Cambridge, MA, USA; ^7^ Center for Patient Safety and Quality Research, Boston Children’s Hospital, Boston, MA, USA; ^8^ Department of Medicine, Obesity Research Center, Boston University School of Medicine, Boston, MA, USA

**Keywords:** topoisomerase I, BRCA1, DNAPK, PTEN, ubiquitin proteasome pathway

## Abstract

Proteasomal degradation of topoisomerase I (topoI) is one of the most remarkable cellular phenomena observed in response to camptothecin (CPT). Importantly, the rate of topoI degradation is linked to CPT resistance. Formation of the topoI-DNA-CPT cleavable complex inhibits DNA re-ligation resulting in DNA-double strand break (DSB). The degradation of topoI marks the first step in the ubiquitin proteasome pathway (UPP) dependent DNA damage response (DDR). Here, we show that the Ku70/Ku80 heterodimer binds with topoI, and that the DNA-dependent protein kinase (DNA-PKcs) phosphorylates topoI on serine 10 (topoI-pS10), which is subsequently ubiquitinated by BRCA1. A higher basal level of topoI-pS10 ensures rapid topoI degradation leading to CPT resistance. Importantly, PTEN regulates DNA-PKcs kinase activity in this pathway and PTEN deletion ensures DNA-PKcs dependent higher topoI-pS10, rapid topoI degradation and CPT resistance.

## INTRODUCTION

Human DNA-topoisomerase I (topoI) reduces DNA supercoiling generated during transcription and replication. The enzyme also plays important roles in chromatin assembly, recombination, and chromosome segregation [1, 2]. TopoI was identified as a specific target for the anticancer drug camptothecin (CPT) [3]. Two CPT analogues, topotecan and irinotecan are used extensively to treat colon, ovarian, pancreatic, breast and small cell lung cancer. Several 3^rd^ generation nano-encapsulated or non-CPT topoI inhibitors are in various phases of clinical development. As a group, CPTs represent one of the most potent classes of anticancer drugs. However, only 13-32% of cancer patients respond to CPTs and mechanisms of resistance are not well understood [4]. Three proposed mechanisms of CPT resistance are: i) lack of drug accumulation due to MDR/ABC transporter, ii) topoI gene mutation, and iii) ubiquitin proteasomal pathway (UPP) mediated degradation of topoI in response to CPT [5]. However, recent studies have shown that CPTs are not MDR substrates, topoI mutations are rare, and they do not account for drug resistance in the patient population [6–10], leaving degradation of topoI as a major potential mechanism of CPT resistance. Other suggested mechanisms for resistance, including regulation of DNA damage response (DDR) pathways, have not been validated, and DNA repair pathways in tumor cells are often defective. Moreover, reports on siRNA library screening and CPT response did not identify a specific gene responsible for resistance [11, 12]. It was also observed that topoI protein levels, mRNA or number of cleavable complexes had no correlation with CPT response [13]. One of the most distinct cellular responses to CPT is the degradation of topoI by the UPP. It was first observed in peripheral leukocytes of patients receiving 9-amino camptothecin [14], and subsequently in multiple cancer cell lines [15]. Additionally, other studies have shown that topoI is degraded by the UPP [16]. These findings that topoI degrades differentially and that the rate of degradation is linked to CPT response was an important observation [17]. This study clearly demonstrated that CPT-induced topoI degradation is not a universal phenomenon and cells representing the same tumor type degrade topoI deferentially. However, the molecular determinants of CPT-induced topoI degradation have not yet been identified. Phosphorylation is another post-translational modification (PTM) that regulates topoI enzyme activity. TopoI was identified as a phosphoprotein and de-phosphorylation of topoI completely eliminates its enzymatic activity [18, 19]. Taken together, phosphorylation and ubiquitination are two well-documented PTMs identified as key regulators of topoI functions. Cross talk between phosphorylation and ubiquitination is well documented [20], and is pertinent to define the molecular fabric of both pathways in order to identify the determinants for CPT resistance.

## RESULTS

### Isolation of topoI interacting protein complex

**Figure 1:**
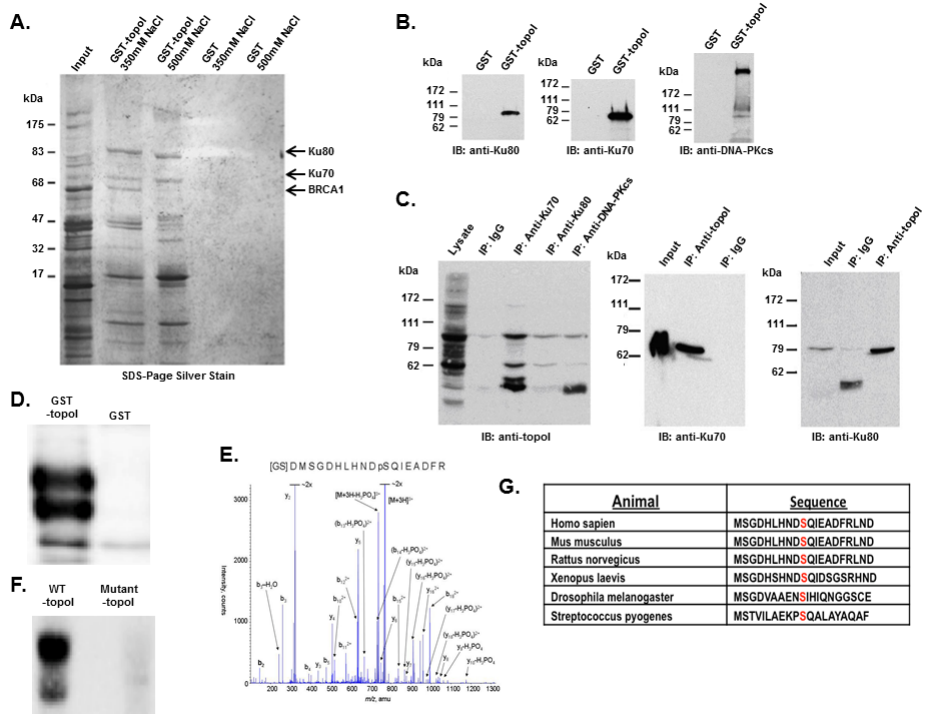
DNA-PK interacts with topoI and DNA-PKcs phosphorylates topoI at S10 **A**. Improvised GST and GST-topoI pull downs were performed with HeLa nuclear extract and proteins associated with GST and GST-topoI were analyzed by SDS-PAGE and silver staining. **B**. GST and GST-topoI pull down experiments with HeLa nuclear extract were performed in small scale and adsorbates were immunoblotted with the indicated antibodies. **C**. HeLa cell lysates were subjected to immunoprecipitation with IgG, anti-Ku70, anti-Ku80, and anti-DNA-PKcs. Hela lysates were also subjected to immunoprecipitation with anti-topoI and immunoprecipitates were immunoblotted with anti-Ku70 (middle panel) and anti-Ku80 (right panel). **D**. DNA-PKcs kinase reactions were performed with GST-topoI and without topoI, and the reaction products were analyzed by SDS-PAGE, coomassie stain and autoradiography. **E**. DNA-PKcs kinase reactions were performed with GST-topoI and the reaction products were analyzed by SDS-PAGE and coomassie staining. GST-topoI was excised, trypsin digested, and analyzed by ESI-MS/MS. The MS/MS spectrum of IMAC enriched phosphopeptide shows fragment ions with topoI-S10 phosphorylation. **F**. DNA-PKcs kinase reactions were performed with GST-topoI-WT and GST-topoI-S10A mutant, and the reaction products were analyzed by SDS-PAGE, coomassie stain and autoradiography. **G**. Sequence alignment of the topoI N-terminal region.

We used an improvised GST-pull down assay to isolate topoI-associated protein complexes [21, 22]. GST-topoI interacting proteins were eluted in buffer containing 350 or 500 mM NaCl and analyzed by SDS-PAGE and silver staining (Figure 1A). The proteins specifically associated with topoI, were in-gel trypsin digested and identified by mass spectrometry. More than nine topoI-interacting proteins were identified including Ku70/Ku80 heterodimer and the BRCT domain of BRCA1. Since the BRCA1 is a 220 KDa protein and the isolated fragment in our assays migrated to 65 kDa, we interpreted the faster migrating band to be a proteolytic fragment of BRCA1. As control, the assays were normalized by GST pull down (Figure 1A).

### TopoI associates with Ku70-Ku80-DNA-PKcs complex in cells

Human topoI, a type 1B enzyme, can relax both negative and positive DNA super coiling by cutting and re-ligating the DNA. However, in the presence of CPTs, the re-ligation step is stalled, and the progressing replication fork during S phase converts the nicked DNA into a DNA-double strand break (DSB) [1, 2]. The Ku70/Ku80 heterodimer recognizes DNA-DSB and targets the DNA-dependent protein kinase catalytic subunit (DNA-PKcs) to the site of strand break to initiate DSB repair by non-homologous end joining (NHEJ) [23]. Thus, our findings provided a potential functional basis for the putative association between topoI and the Ku70/80 heterodimer. To further understand the association between topoI and the Ku70/Ku80 heterodimer, GST and GST-topoI were separately bound to sepharose beads, incubated with HeLa cell lysates and adsorbates were immunoblotted with antibodies to Ku80, Ku70 or DNA-PKcs. The results demonstrate that in contrast to GST, Ku80 (Figure 1B left panel), Ku70 (Figure 1B middle panel) and DNA-PKcs (Figure 1B right panel) proteins were identified in the GST-topoI adsorbates. To rule out the possibility of DNA strand-mediated association, we have added ethidium bromide in the binding and wash buffer. To validate further these associations, immunoblot analysis of topoI immunoprecipitates demonstrated co-precipitation of a Ku70-Ku80-DNA-PKcs complex with topoI (Figure 1C, left panel). Similar results were obtained in the reciprocal experiment in which anti-topoI immunoprecipitates were analyzed by immunoblotting with anti-Ku70 (Figure 1C, middle panel) and anti-Ku80 (Figure 1C, right panel). Taken together, these findings strongly indicated that topoI is in the complex of Ku70-Ku80-DNA-PKcs.

### TopoI Serine 10 is phosphorylated by DNA-PKcs

In the response to DNA-DSBs, DNA-PKcs forms a complex with Ku70/Ku80 heterodimer and the complex is referred to as DNA-PK. To understand the cellular function of topoI-DNA-PK association, we first asked if topoI is phosphorylated by DNA-PK. GST-topoI was incubated with purified DNA-PK and the kinase reaction products were analyzed by SDS-PAGE and autoradiography. The results demonstrated that DNA-PK phosphorylates topoI *in vitro* (Figure 1D). To identify the site of phosphorylation, topoI protein was digested and analyzed by mass spectrometry. Analysis of immobilized metal affinity chromatography (IMAC) enriched phosphopeptide resulted in the identification of [GSD]MSGDHLHNDpSQIEADFR peptide. This trypsin digested GST-topoI peptide contains three amino acids [GSD] from the GST protein, while the remaining 17 amino acids were from the N-terminus of topoI. The y and b ion series confirmed the sequence of peptides, as well as the phosphorylation of Serine 10 of topoI (Figure 1E). No phosphorylation was observed when a mutant (S10♢A) topoI was incubated with the purified DNA-PK (Figure 1F and Supplementary Figure S1A). These findings indicated that topoI-S10 is the only site that is phosphorylated by DNA-PK *in vitro*. As one measure of the significance of topoI S10 phosphorylation, the conservation of aa 1-20 was determined by BLAST analysis. Furthermore, in this context, the sequence homology surrounding topoI S10 and the SQ motif (the consensus motif for DNA-PKcs phosphorylation) is highly conserved (Figure 1G).

### CPT-induced topoI degradation requires DNA-PKcs dependent phosphorylation of topoI-S10

**Figure 2:**
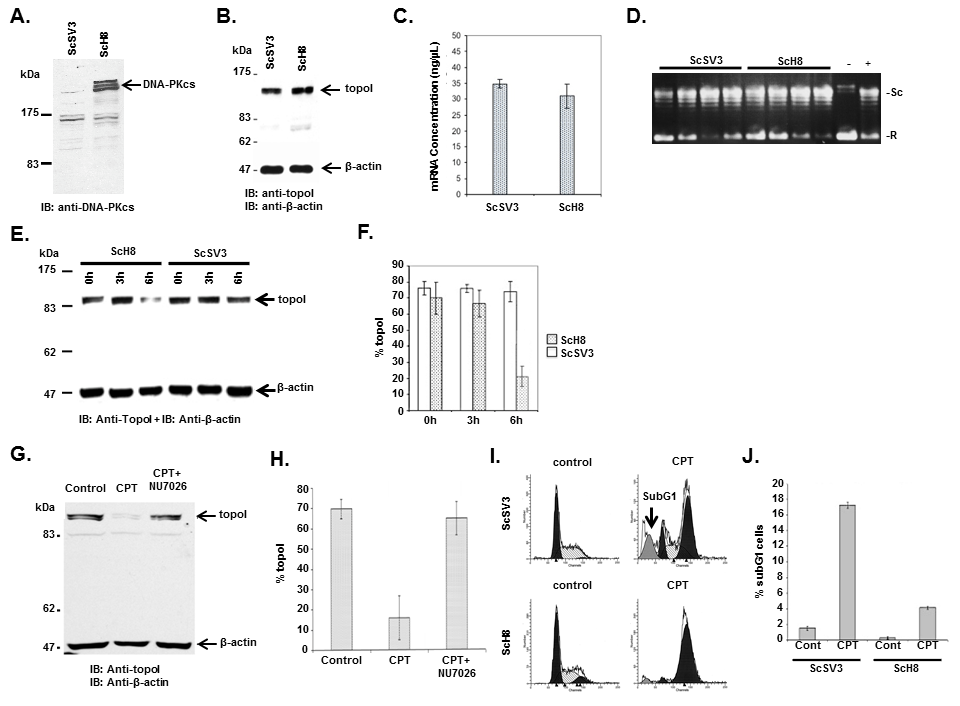
DNA-PKcs dependent topoI-S10 phosphorylation is required for topoI degradation in response to CPT **A**. ScSv3 and ScH8 cells lysates were analyzed by immunoblot with anti-DNA-PKcs. **B**. ScSv3 and ScH8 cell lysates were analyzed by immunoblot analysis with anti-topoI and anti-β-actin. **C**. ScSv3 and ScH8 cells were harvested and mRNA was extracted. TopoI mRNA was quantitatively analyzed by RT-PCR. **D**. TopoI activity assays were performed by incubating supercoiled (SC) DNA in the presence of nuclear extract from ScSV3 and ScH8 cells, and subsequently separated by electrophoresis in the presence of ethidium bromide. The larger proportion of relaxed (R) DNA compared to supercoiled DNA (SC) indicates higher activity of topoI. **E**. ScH8 and ScSV3 cells were treated with 25µM irinotecan and harvested at 3h and 6h. Cells lysates were immunoblotted with anti-topoI and anti-β-actin. **F**. Results of three independent experiments described in E were quantitatively analyzed (mean +/- SD). **G**. HeLa cells were treated with irinotecan and irinotecan+DNA-PK inhibitor; NU7026 for six hours. Cell lysates were analyzed by immunoblotting with anti-topoI and anti-β-actin. Non-treated cells were also analyzed. **H**. Results of three independent experiments described in I were quantitatively analyzed by densitometry (mean +/- SD). **I**. ScH8 and ScSV3 were treated with 20nM irinotecan for 72 hours and analyzed for DNA content /sub G1 analysis (indicated by arrow) by FACS. ScSV3 control and treated cells (upper panel) and ScH8 control and treated cells (lower panel). **J**. Results of three independent experiments described in G were quantitatively analyzed (mean +/- SD).

To determine the role of topoI-S10 phosphorylation on topoI regulation, we assessed topoI protein and mRNA level as well as its activity in ScSV3-MEF (DNA-PKcs−/−) cells and kinase reconstituted ScH8-MEF (DNA-PKcs+/+) cells [24]. Immunoblot analysis of ScSV3 and ScH8 cells demonstrated deletion of DNA-PKcs protein in ScSV3 cells as expected (Figure 2A). We then asked if there is any functional difference in topoI activity in these cell lines. The results demonstrate no difference in topoI proteins (Figure 2B), mRNA or enzyme activity (Figure 2C and 2D). Importantly, the cellular response to CPT was remarkably different in these cell lines. Our results demonstrated that in response to CPT, the topoI protein was rapidly degraded in DNA-PKcs proficient ScH8 cells, while no or minimal topoI degradation was observed in DNA-PKcs deficient ScSV3 cells (Figure 2E and 2F). To substantiate the evidence, we next used a inhibitor of DNA-PKcs (NU7026) and determined that topoI is not degraded in response to CPT in the presence of NU7026 (Figure 2G and 2H). The rate of topoI degradation has been linked to cellular sensitivity to CPTs [17]. ScSV3 and ScH8 cells were treated with irinotecan and subjected to sub G1 analysis after 72 hours. The results clearly demonstrate that DNA-PKcs deficient cells are very sensitive to irinotecan (Figure 2I and 2J). We next determined cellular sensitivity to SN-38 (active metabolite of irinotecan) in MO59J (DNA-PKcs−/−) and MO59K (DNA-PKcs +/+) cells. The results showed that in contrast to MO59K, MO59J cells are sensitive to SN-38 (Supplementary Figure S1B). We also determined the basal topoI-pS10 level in DNA-PKcs-positive and DNA-PKcs deficient cells and their potential link to CPT response. Immunofluorescence analysis with anti-topoI-pS10 demonstrated higher basal level of topoI-pS10 in MO59K cells (Supplementary Figure S1C). Taken together, these findings indicated that DNA-PKcs dependent phosphorylation of topoI-S10 is critical for CPT-induced topoI degradation and a higher basal level of topoI-pS10 ensures rapid degradation of topoI and CPT resistance.

### BRCA1 binds and ubiquitinates topoI for proteasomal degradation

**Figure 3:**
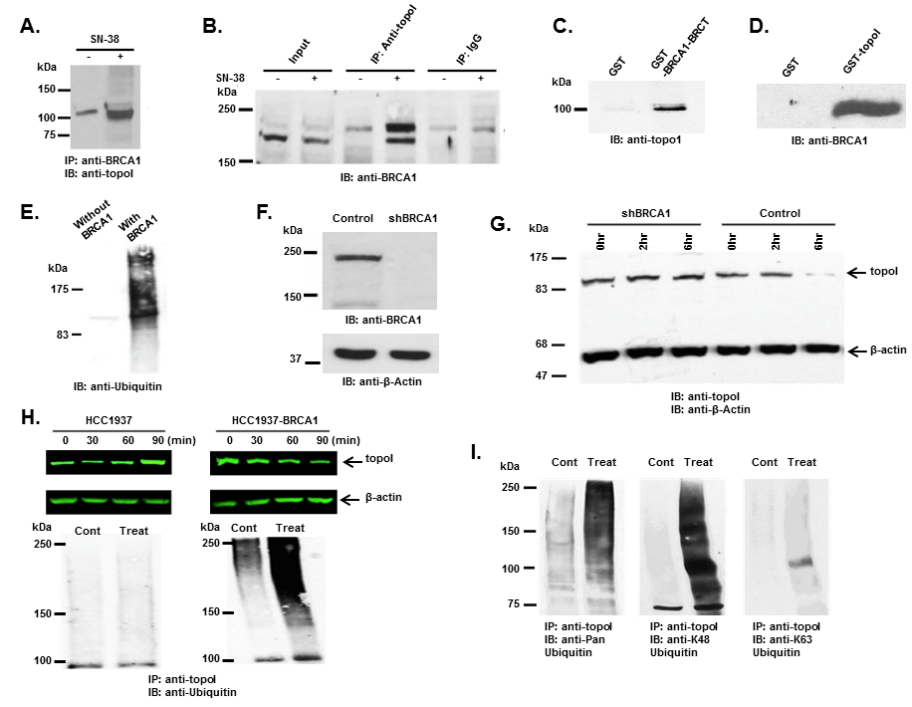
BRCA1 associates and ubiquitinates topoI **A**. Control and CPT-treated (5 μM SN-38) HCT-15 cells were lysed and subjected to immunoprecipitation with anti-BRCA1. Immunoprecipitates were immunoblotted with anti-topoI. **B**. Control and CPT-treated (5 μM SN-38) HCT-15 cell lysates were subjected to immunoprecipitation with anti-topoI or IgG (control) and immunoprecipitates were immunoblotted with anti-BRCA1. **C**. GST and GST-BRCA1-BRCT were incubated with HCT-15 cell lysate. After extensive washing the adsorbates were analyzed by immunoblotting with anti-topoI. **D**. GST and GST- topoI (aa1-210) were incubated with HCT-15 cell lysates. After extensive wash the adsorbates were analyzed by immunoblot analysis with anti-BRCA1. **E**. GST-topoI was phosphorylated with DNA-PK and then incubated with purified E1, UbcH5c (E2), and BRCA1/BARD1 (E3) and without BRCA1/BARD1. The reaction products were immunoblotted with anti-ubiquitin. **F**. BT-474 cells were transduced with BRCA1 silencing lentivirus (shBRCA1) and scrambled sequence lentivirus (as control). Control and shBRCA1 cells lysates were analyzed by immunoblotting with anti-BRCA1 and anti-β-actin. **G**. Control and shBRCA1 BT-474 cells were treated with 5 μM SN-38 for 2h and 6h and the cell lysates were immunoblotted with anti-topoI and anti-β-actin. **H**. HCC1937 and HCC1937-BRCA1, cells were treated with 5 μM SN-38 for 30, 60 and 90 min. Cell lysates were immunoblotted with anti-topoI (upper panel) and β-actin (middle panel). Cell lysates were also subjected to immunoprecipitation with anti-topoI and immunoprecipitates were immunoblotted with anti-ubiquitin (lower panel). **I**. HCC1937-BRCA1 cells were treated with 5 μM SN-38 and cell lysates were subjected to immunoprecipitation with anti-topoI. The immunoprecipitates were analyzed by immunoblot with the indicated antibodies.

In our GST-topoI pull down experiment, a protein with a molecular mass of approximately 65 kDa was identified by MS/MS analysis as BRCA1. The BRCA1 polypeptide is approximately 220 kDa, however, in our pulldown experiment, a BRCA1 proteolytic fragment containing the BRCT domain associated with topoI was identified. To better understand the UPP-mediated topoI degradation in the response to CPT, we asked if phosphorylated topoI is an E3 ligase substrate of BRCA1. Co-immunoprecipitation and direct binding experiments were performed to further understand the interaction between topoI and BRCA1. Our results demonstrated that topoI associate with BRCA1 and that this complex formation is enhanced in cells that are treated with CPT (Figure 3A and 3B). Direct binding experiments using GST pull down analysis also demonstrated that the topoI amino terminal region domain binds directly with BRCA1-BRCT domain (Figure 3C and 3D)

To determine whether topoI is ubiquitinated by BRCA1 in response to CPT-induced DSB formation, we first determined whether topoI is ubiquitinated by BRCA1 *in vitro*. GST-topoI phosphorylated by DNA-PK was incubated with the BRCA1/BARD1 heterodimer in the presence of ubiquitin-activating enzyme E1, ubiquitin conjugating enzyme E2 (UbcH5c) and ATP in ubiquitination buffer. The reaction products were analyzed by immunoblotting with anti-ubiquitin. The results demonstrated that BRCA1 ubiquitinates topoI in vitro (Figure 3E). We then asked if topoI is ubiquitinated by BRCA1 in cells. We silenced BRCA1 in BT-474 by shRNA (shBRCA1-BT-474) (Figure 3F) and asked if topoI degradation was inhibited in BRCA1 silenced cells. The results clearly demonstrated that topoI is degraded in the response to CPT in BT-474 wild-type cells. However, no topoI degradation was observed in shBRCA1-BT-474 cells after 6 hours of CPT treatment (Figure 3G). We extended the study in E3 ligase-deficient HCC1937 cells with BRCA1 deletion and HCC1937 cells stably expressing WT BRCA1 (HCC1937-BRCA1) (Supplementary Figure S2) and determined CPT-induced topoI degradation. Our findings demonstrate that topoI is not degraded in HCC1937 cells. However, HCC1937-BRCA1 cells efficiently degraded topoI in the response to CPT (Figure 3H, upper panel). We also determined the ubiquitination status of topoI in HCC1937 and HCC1937-BRCA1 cells. In contrast to HCC1937, pronounced topoI ubiquitination was observed in HCC1937-BRCA1 cells treated with CPT (Figure 3H, lower panel). We used a K-48 specific ubiquitin antibody to determine the type of topoI ubiquitination and the results indicated that ubiquitin is attached to topoI *via* lysine 48 for proteasomal degradation (Figure 3I). Taken together, these findings demonstrate that CPT induced topoI degradation is UPP mediated and topoI is ubiquitinated by BRCA1.

### CPT-induced rate of topoI degradation determines the CPT response

**Figure 4:**
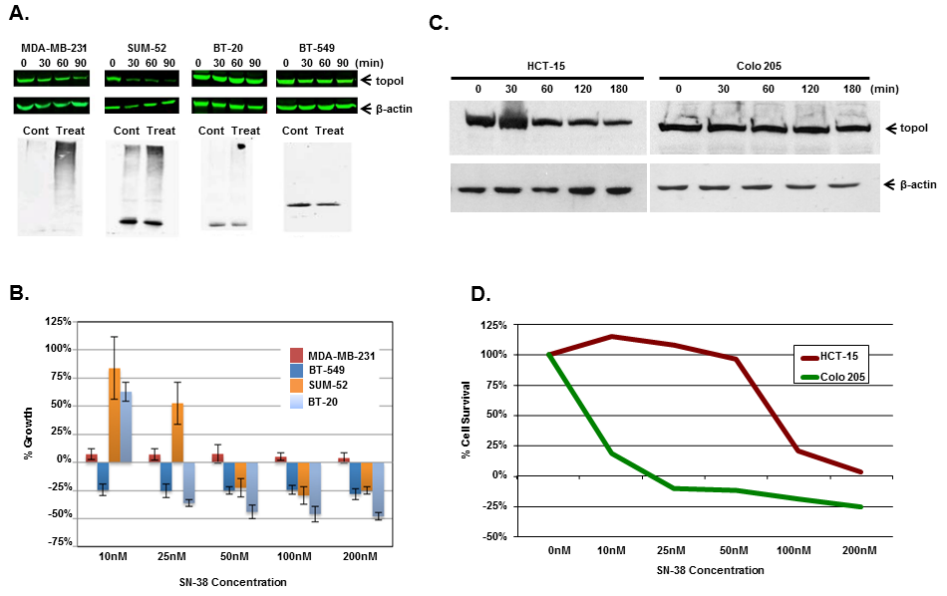
Rate of topoI degradation determines the cellular response to CPT in triple negative breast cancer cells and colorectal cancer cells **A**. Triple negative breast cancer cell lines, MDA-MB-231, SUM-52, BT-20 and BT-549 cells, were treated with 5 μM SN-38 for 30, 60 and 90 min. Cell lysates were immunoblotted with anti-topoI (upper panel) and β-actin (middle panel) antibody. Cell lysates were also subjected to immunoprecipitation with anti-topoI and immunoprecipitates were immunoblotted with anti-ubiquitin (lower panel). **B**. MDA-MB-231, SUM-52, BT-20 and BT-549 cells were treated with different concentrations of SN-38 and were analyzed for percent growth by Celigo direct cell counting after 72 hours (mean +/- SD). **C**. Colon cancer cell lines, Colo 205 and HCT-15 cells, were treated with 20µM Irinotecan and the cell lysates were immunoblotted with anti-topoI and β-actin antibody. **D**. Colo 205 (green) and HCT-15 (red) cells were plated in a 96 well plate and treated with different concentrations of irinotecan for 72 hours. Cell viability was determined by MTT assay.

Multiple cell lines (triple negative breast cancer; TNBC; colorectal cancer; CRC and small cell lung cancer) were used to determined the rate of topoI degradation in the response to CPT. The data clearly demonstrates that the cells that degrade topoI rapidly are resistant to CPT (Figure 4 and Supplementary Figure S3). In TNBC cells, we determined the status of topoI ubiquitination, rate of degradation and sensitivity to CPT. Results demonstrate that topoI is ubiquitinated only in those cell lines that degrade topoI (MDA-MB-231 and SUM-52), while no ubiquitination was observed in BT-20 and BT-549 (Figure 4A). Also, the data strongly indicate that the cells that degrade topoI rapidly are resistant to CPT while cells that fail to degrade topoI are sensitive to CPT (Figure 4B).

### TopoI degradation and drug sensitivity are linked to topoI-S10 phosphorylation

**Figure 5:**
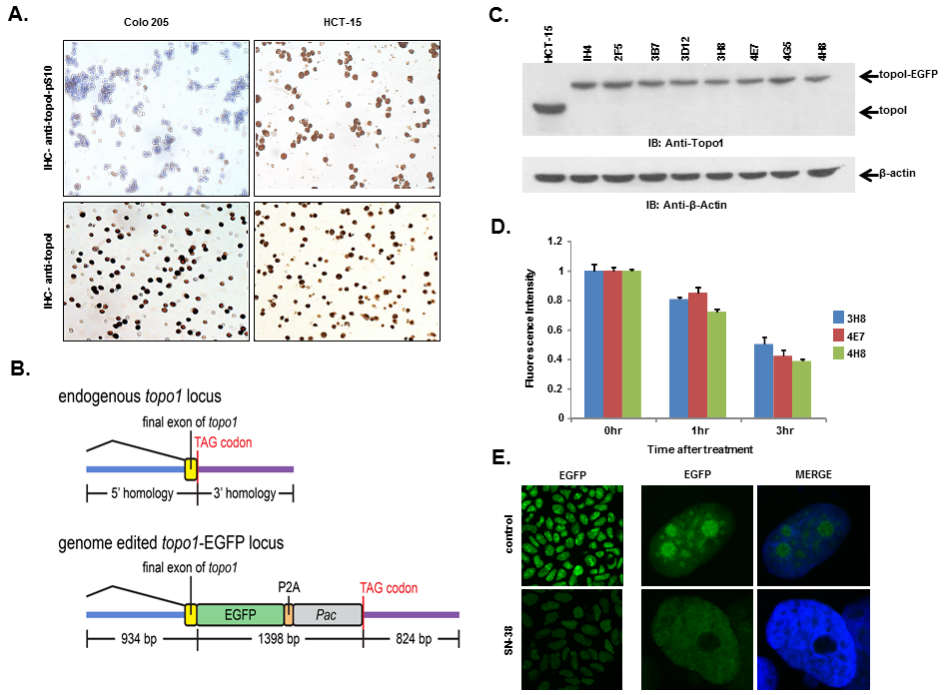
HCT-15 cells have higher basal level of topoI-pS10 and generating topoI-EGFP fusion cells **A**. Colo 205 and HCT-15 cell pellets were fixed and subsequently embedded in paraffin. Slides were cut from the cell pellet and IHC staining with anti-topoI-pS10 (upper panel) and anti-topoI (lower panel) was performed. **B**. A SpCas9-VQR variant plasmid and a sgRNA expression plasmid targeted to the *hTOP1*stop codon were co-transfected with a homologous recombination donor plasmid in HCT-15 cells to generate topoI-EGFP fusion protein expressing cell lines. **C**. Single EGFP positive cells were sorted and grown, and several clones were selected and characterized by western-blot. Cells lysates were subjected to immunoblot analysis with anti-topoI (upper panel) and anti-β-actin (lower panel). **D**. Three clones (3H8, 4E7 and 4H8) were further characterized by determining topoI degradation. Cells were treated with 2.5μM SN38 for 3h and EGFP florescence intensity was quantitatively analyzed by plate reader. **E**. Cells from clone 4E7 (HCT-15 4E7) were treated with 2.5μM SN38 (active metabolite of irinotecan) for 3h. Cells were visualized by using a Leica S5 confocal microscope.

We, as well as others, have shown that rapid degradation of topoI leads to CPT resistance [17]. Here, we have also demonstrated that DNA-PK mediated phosphorylation of topoI-pS10 is critical for CPT induced topoI degradation. We next asked if topoI-pS10 levels predict rapid degradation of topoI and CPT resistance in CRC cells. In response to CPT treatment, topoI was degraded rapidly in HCT-15 colon carcinoma cells while little, if any, degradation was observed in Colo 205 cells. Cell viability data also indicated that HCT-15 cells are at least ten fold more resistant to CPT than Colo 205 cells (Figure 4C and 4D). We then asked if the rate of degradation is linked to topoI-pS10 level. Immunohistochemistry (IHC) with our mouse monoclonal antibody (Clone 1C1.H5.H7) demonstrated a strong topoI-pS10 nuclear staining in HCT-15 cells. In contrast, few topoI-pS10 positive cells were seen in Colo 205 cells (Figure 5A upper panel). IHC assays were also performed using anti-topoI and data demonstrates that topoI protein level is similar in Colo 205 and HCT-15 cells (Figure 5A, lower panel). These results were consistent with higher topoI-pS10 indicates rapid topoI degradation and CPT resistance.

### Developing a gene edited cell line to quantitatively analyze CPT induced topoI degradation

To summarize, our studies have revealed that, i) topoI is rapidly degraded in CPT-resistant cells, ii) higher basal levels of topoI-pS10 ensures rapid degradation of topoI and thus cause CPT resistance and iii) that DNA-PK phosphorylates topoI at S10. To better understand CPT resistance mechanisms, it therefore became imperative to understand the upstream regulation of DNA-PKcs. To visualize topoI degradation in the response to CPT in real time, we fluorescently tagged topoI in HCT-15 cells. An endogenous fusion to EGFP was generated using CRISPR/Cas9 genome editing in the presence of a homologous recombination donor that resulted in integration of EGFP at the C-terminus of the *hTOP1* gene (Figure 5B). Following puromycin selection, single EGFP positive cells were sorted and grown, sequence analysis confirmed the integration of EGFP at the C-terminal end of the *hTOP1* gene (data not shown). A change in the molecular mass of approximately 24kDa indicates the integration of EGFP into topoI (Figure 5C). Clonal topoI-EGFP cell lines enabled us to achieve two specific goals: first, to quantitatively analyze topoI degradation in the response to CPT, and secondly, it provided an efficient assay to identify the potential upstream regulators of DNA-PKcs.

It has also been established that CPT induces rapid nucleolar clearance of topoI [25]. We therefore asked if our genetically-edited cells have these functional attributes. Three topoI-EGFP integrated HCT-15 cell clones, 3H8, 4E7 and 4H8 were treated with 2.5μM SN-38 and florescence intensity was quantitatively-analyzed (Figure 5D). Nuclear re-localization of topoI-EGFP was observed by confocal microscopy, which was consistent to the previous report [26]. A significant reduction in topoI-EGFP signal and rapid nuclear re-localization of topoI-EGFP in CPT-treated cells was observed in all the clones (Figure 5E). To further confirm topoI function in genome edited cell lines, we determined the CPT sensitivity by clonogenic survival assays. As expected, a comparative analysis indicated that HCT-15 cells are more resistant to HCT-116 cells (both genetically edited). (Supplementary Figure S4). These findings confirmed that the topoI-EGFP fusion protein follows the functional pathway of WT topoI protein in genetically edited cells. Based on this and other data, we selected clone 4E7 for our siRNA library screen experiments.

### iRNA library screen to identify upstream regulators of DNA-PKcs in response to CPT

We have demonstrated that a higher basal level of topoI-pS10 ensures topoI ubiquitination and rapid proteasomal degradation of topoI, leading to CPT resistance. These findings indicated that in these cells, DNA-PKcs remains active and ensures a higher basal level of topoI-pS10. Phosphorylation and de-phosphorylation have been a primary mechanism of DNA-PKcs activation and inactivation. DNA-PKcs is activated by recruitment to DSBs, *via* Ku70/80. Once activated, autophosphorylation causes subsequent inactivation of DNA-PK and dissociation from DSB ends [27, 28]. Protein phosphatases have been shown to interact with DNA-PKcs and could serve as potential upstream regulators of DNA-PKcs [29]. However little is known regarding this aspect in the literature and the mechanism is not well defined. Using HCT-15 4E7 cells, we asked if nuclear phosphatases regulate CPT-induced topoI degradation. We preformed an siRNA library screen of 56 reported nuclear phosphatases, and phosphatase silenced cells were treated with CPT. GAPDH siRNA was used as a control. Silencing of nuclear phosphatase genes affected topoI degradation (Supplementary Figure S5). Based on the data from the first screen, we selected 9 genes that enhanced topoI degradation the most, as well as the 9 genes that contributed least. Thus, in the second phase of the siRNA library screen, we used only 19 selected genes (including one control) to determine the effect of specific phosphatases in CPT-induced topoI degradation.

**Figure 6:**
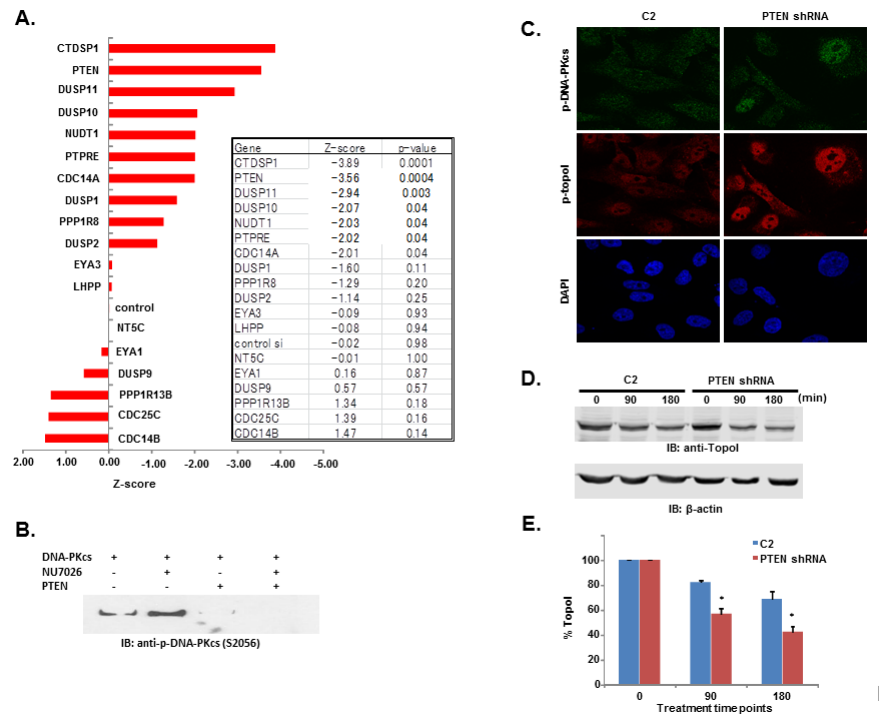
DNA-PKcs dependent topoI-S10 phosphorylation is regulated by PTEN **A**. A siRNA screen was performed in two phases, in the first phase 56 nuclear phosphatases were silenced using siRNA library (GE-Dharmacon). Phosphatases were silenced in HCT-15 4E7 cells and cells were treated with 2.5μM SN-38 for 4h. EGFP florescence intensity, indicating rate of topoI degradation, was analyzed using a plate reader. Multiple silencing experiments were performed using the 18 genes that most affected the topoI-EGFP degradation. The most and least significant phosphatases are listed. Bar graph shows the Z-score of the most and the least significant effect on topoI degradation in response to CPT. CTDSP1 and PTEN were identified as the most significant phosphatases inhibiting topoI degradation. **B**. DNA-PKcs kinase reactions were performed with or without DNA-PKcs inhibitor and PTEN. The reaction products were analyzed by immunoblot analysis with a phosphospecific antibody to DNA-PKcs-S2056. **C**. C2 and PTEN shRNA cells were stained by immunofluorescence with phosphospecific anti-DNA-PKcs-S2056 and anti-topoI-pS10 antibodies. **D**. To determine the rate of topoI degradation, C2 and PTEN shRNA cells were treated with 2.5μM SN38 for 90 and 180 min. Cell lysates were immunoblotted with anti-topoI (upper panel) and anti-β-actin (lower panel). **E**. Quantitative estimation of topoI protein in C2 and PTEN shRNA cells treated with SN-38 in Figure 6D. * *P* < 0.01. **F**. C2 and PTEN shRNA cells were treated with 2.5µM SN-38 for 90 and 180min. The TopoI protein level was determined by immunofluorescence staining with anti-topoI (red). **G**. Graph shows a quantitative estimation of topoI immunofluorescence labeled with topoI in C2 and PTEN shRNA cells in Figure 6F. * *P* < 0.01. **H**. C2 and PTEN shRNA cells were treated with various concentrations of SN-38 and clonogenic survival assays were performed. Colonies were counted and relative number of colonies was determined. **I**. C2 and PTEN shRNA cells were treated with SN-38 at indicated concentration for 24 hours. After 72 hours of incubation, cells were harvested and analyzed for DNA content /sub G1 analysis by FACS. Results of three independent experiments were quantitatively analyzed (mean +/- SD). ** *P* < 0.0001.

Relative intensity of GFP fluorescence was measured after 4 hours of CPT treatment and the mean level of GFP fluorescence was also determined. We then calculated the z score using the formula (mean gene-mean control)/sd (control). Bonferonni corrections were used to determine significance threshold (*p* = 0.0025). Genes showed both positive and negative z-scores. The positive z-scores indicated that topoI degradation was stabilized after silencing the specific gene. Silencing of CDC 14B showed the least topoI degradation although not significantly different compared to control (Figure 6A). Silencing of several genes increased the degradation of topoI, indicated by negative z-scores. Genes DUSP10, NUDT1 and PPTRE showed marginally significant reduction (*p* = 0.04), and gene DUSP11 showed more pronounced reduction beyond control (*p* = 0.003). Silencing of genes CTDSP1 and PTEN showed a statistically significant (Bonferonni corrected threshold) reduction in GFP florescence relative to control (*p* < 0.0001) (Figure 6A).

### PTEN dephosphorylates DNA-PKcs and PTEN deficient cells have higher basal level of topoI-pS10

DNA-PKcs is activated and inactivated by phosphorylation. Although the full effects of phosphorylation and kinase inactivation remain to be understood, autophosphorylation of serine 2056 (pS2056) has been shown to be a reliable indicator of DNA-PKcs activation [30]. Our siRNA screen data indicates that silencing PTEN significantly enhanced topoI degradation in response to CPT and that DNA-PKcs dependent topoI-pS10 is higher in these cells. We first asked whether PTEN dephosphorylates DNA-PKcs. A DNA-PKcs autophosphorylation kinase assay was performed, and at the completion of the assay purified PTEN was added. Our results demonstrate that addition of PTEN resulted in dephosphorylation at S2056 (Figure 6B). We used a MDA-MB-231 cell line with stably silenced PTEN by shRNA (PTEN shRNA) (Supplementary Figure S6A) and control C2 cells and asked if there is any difference in the basal level of phospho-DNA-PKcs and topoI-pS10. PTEN shRNA cells showed a higher basal level of DNA-PKcs-S2056 and topoI-S10 phosphorylation (Figure 6C and Supplementary Figure S6B). Since we have demonstrated that higher basal level ensures rapid degradation of topoI in response to CPT, we next asked whether this is controlled by PTEN. C2 and PTEN shRNA cells were treated with SN-38 and cell lysates were immunoblotted with an anti-topoI antibody. PTEN-deficient cells with higher levels of phospho DNA-PKcs and phospho-topoI showed a rapid rate of topoI degradation compared to control cells (Figure 6D and 6E). These results were further confirmed by the immunostaining of cells with an anti-topoI antibody (Figure 6F and 6G). One of most distinct functional validations of topoI degradation is the cellular sensitivity to CPT. We asked if PTEN shRNA cells that rapidly degrade topoI are resistant to CPT. Clonogenic survival assays and subG1 analysis by FACS were performed and our data indicates that PTEN shRNA cells are indeed more resistant to CPT (Figure 6H and 6I).

## DISCUSSION

Human topoI, a type IB enzyme, reduces DNA supercoiling by cutting and re-ligation of a single DNA strand. The cutting and re-ligation of DNA is achieved by two transesterification reactions. In the first reaction, topoI-723 tyrosyl attacks the DNA phosphate group causing a DNA strand break and tyrosyl oxygen is covalently linked to the 3’ phosphate group of DNA. The second transesterification causes the reversal of the first reaction leading to rejoining of the broken strand. A controlled rotation, between the cutting and the re-ligation reduces the supercoiling [2]. However, in the presence of CPT, the re-ligation step is blocked, resulting in an accumulation of covalent reaction intermediates, referred to as the cleavable complex. The formation of cleavable complexes leads to stalled replication forks, and ultimately DSBs [1]. The crystal structure of the topoI-DNA-CPT complex indicates that topotecan (a CPT analogue) mimics a DNA base pair and binds to the complex between the DNA bases of both-strands at the DNA cleavage site [31]. This complex block not only the re-ligation step, but also access to the 3’-termini required for loading of protein complexes for DNA repair. In human cells DSBs are repaired by two main pathways, NHEJ, which is initiated by recruitment of Ku and DNA-PKcs and is active throughout G1, S and G2 [32] and homologous recombination (HR), which is active only in S and G2. HR -mediated repair begins with resection of the DNA-DSB by MRN complex to create a long stretch of single stranded (ss) DNA with a 3’end for recruitment of RPA which initiates recruitment of Rad51 for HR [33].

CPT-induced DNA-DSB is unique and the precise mechanism of early DDR is not understood. The strand breaks are single-ended double strand breaks, topoI polypeptide is linked to 3’ of the broken strand and broken strand with repair available 5’ is a collapsed replication fork. Pathway choice, in single-ended double-strand breaks, between most prevalent NHEJ and S-phase specific HR-mediated repair is yet to be defined. The MRN complex plays a central role in pathway determination. Mre11 endonuclease activity nicks adjacent to DSB. Subsequently, resection proceeds in both directions *via* Mre11 3’-5’ and Exo1/BLM 5’-3’ exonuclease activity [34]. However, this model is based on XRT induced single-ended DNA-DSB. This model does not explain the resolution of complexity rendered by CPT induced single-ended DSB, particularly how topoI is removed and the collapsed replication fork is restored. In S. cerevisiae, Mre11 mutant sensitivity in the response to CPT is suppressed in Ku70 deleted cells, indicating interplay between these two proteins at single ended DSBs. This interplay determines the extent of Exo1- dependent resection at the 5’ of the broken strand [35]. DT40 chicken cells deficient in Ku80 are resistant to CPT [36, 37]. Similarly, in CHO cells, Ku80 deficient cells were not CPT sensitive [38]. However, in this study, Shin et al. used high concentration CPT (1μM) for short duration (only 30 minutes) and relatively low concentration (100 nM CPT) for longer duration. These concentrations do not conclusively address the role of Ku80 in CPT-induced DDR. We have demonstrated that topoI associates with DNA-PK and DNA-PKcs phosphorylates topoI for ubiquitination and proteosomal degradation. Thus, our work indicates that NHEJ sensor complex stabilizes the broken strand and initiates the removal of topoI for repair. Our findings are in concert with reports on the activation of DNA-PKcs by CPT [39, 40]. The role of DNA-PK in CPT induced DNA-DSB is further supported by our data demonstrating that DNA-PKcs deficient cells are sensitive to CPT (Figure 2I, 2J and Supplementary Figure S1B).

BRCA1 plays a critical role in genome maintenance [41]. Critical to its many functions is its association with various protein complexes involved in DSB repair [42]. However, the precise mechanism by which BRCA1 functions in DSB repair is still not well understood. Here, we use three different approaches (highly purified *in vitro* ubiquitination assays, silencing of BRCA1, and expressing BRCA1-WT in HCC-1937 cells) to demonstrate that BRCA1 ubiquitinates topoI *in vitro* and in cells. In an effort to understand BRCA1 E3 ligase function in DNA-DSB, Reid et al. generated isogenic clones of embryonic stem cells with BRCA1-WT, I26A (a mutant that only impairs BRCA1 E3 ligase function and not BRCA1-BARD1 interaction), and a BRCA1 deletion mutant that affects BRCA1 DDR function significantly. Using mitomycin C (MMC) and ionizing radiation (IR), they demonstrated that E3 ligase deficient cells are not sensitized to these agents, and that the rates of homology directed DNA-DSB repair are similar [43]. In striking contrast, BRCA1-E3 ligase inactivation impairs the DDR when CPT is used as the DNA damaging agent in DT40 chicken cells. In these cells, V26A abrogates the E3 ligase activity of BRCA1. Cell viability, Rad51 foci formation and sister chromatid exchange (SEC) rates were all similar in BRCA1 WT and V26A cells in response to MMC. However, V26A mutant cells were very sensitive to CPT, with a sharp reduction in RAD51 foci formation and SEC frequency [44]. Collectively, these studies demonstrate that BRCA1 E3 ligase function is critical only for CPT-induced DDR. Our findings not only support these observations, but also define the mechanism for this specificity. Specifically, we show that HCC1937 cells, with BRCA1 deletion, fail to degrade topoI and are sensitive to CPT while HCC1937 cells reconstituted with BRCA1 WT degraded topoI in response to CPT and are resistant to CPT. Significantly, we show that CPT induced topoI ubiquitination and degradation is directly linked to CPT response as demonstrated in four TNBC cells. Taken together, we conclude that BRCA1 is the E3 ligase for topoI, and the rate of topoI proteasomal degradation determines CPT response.

Our findings demonstrating CPT-induced proteasomal degradation of topoI is in agreement with published literature. The literature also demonstrates that topoI is degraded differentially in cancer cells and CPT response depends on the rate of topoI degradation. We have replicated these findings in ten cancer cell lines representing CRC, TNBC and SCLC (Figure 3 and Supplementary Figure S3). However, one of our most important findings is to demonstrate that the basal level of topoI-pS10 is critical for topoI ubiquitination. The implications of these findings are as follows: first, we understand the molecular mechanism of topoI ubiquitination and more importantly topoI-pS10 is a molecular determinant for CPT-induced rate of topoI degradation and drug response. The higher basal level of topoI-pS10 predicts the rapid rate of topoI degradation and cellular resistance to CPT. In contrast, cells with lower levels of topoI-pS10 fail to degrade topoI, resulting in impaired DDR and cell death.

We have demonstrated higher basal cellular level of topoI-pS10 in cancer cell lines, and various tumor types. These findings also indicate that the cells with higher basal level of topoI-pS10 have higher DNA-PKcs kinase activity (inferred from increased DNA-PKcs S2056 phosphorylation). There are three potential mechanisms for DNA-PKcs regulation: DDR related activation and inactivation, changes in protein expression and upstream phosphatases. Activation of DNA-PKcs in response to DSBs is relatively well understood and higher levels of DNA-PKcs expression have been reported in various tumor types including CRC, linking them to tumor pathogenesis and tumor progression, [45–49]. DNA-PKcs has also been identified as selective regulator of transcription leading to prostate cancer progression and metastasis. DNA-PKcs is up regulated and activated significantly in advanced disease. This activation was independent of its DDR activities and it correlated with tumor metastasis, recurrence and poor survival [50]. IHC analysis of tumor microarray slides representing CRC, breast, ovarian and SCLC shows differential topoI-pS10 independent of stage of the tumor (Supplementary Figure S7). This suggests that the topoI-pS10 level depends on DNA-PKcs non-DDR activation and also on tumor progression dependent higher DNA-PKcs protein level. Protein phosphatases have been suggested as the potential upstream regulators of DNA-PKcs [29] but the mechanism is not well defined. Here, we identify PTEN as a novel potential DNA-PKcs regulatory phosphatase. PTEN is a tumor suppressor gene that possesses both protein and lipid phosphatase activity. The lipid phosphatase activity is linked to tumor suppressor, whereas the protein phosphatase activity is regulatory in nature [51]. PTEN is a dual phosphatase, as it can dephosphorylate protein and peptide substrates phosphorylated on serine, threonine and tyrosine [52]. Our library screen data was confirmed by using stably PTEN silenced MDA-MB-231 cells. PTEN shRNA cells had a higher level of phosphorylated DNA-PKcs (S2056) and topoI-pS10. Thus, various lines of evidence demonstrated that PTEN is one of the upstream effectors in DNA-PKcs dependent topoI proteasomal degradation pathway. This observation was further confirmed by our findings that PTEN phosphatase-deficient cells degrade topoI rapidly and are resistant to CPTs. Further studies would be required to understand a potential universal link between PTEN tumor suppressor mechanisms and drug resistance. However, our data indicates that deletion of tumor suppressor PTEN imparts CPT resistance, enhances the survival and ultimately tumor progression.

**Figure 7:**
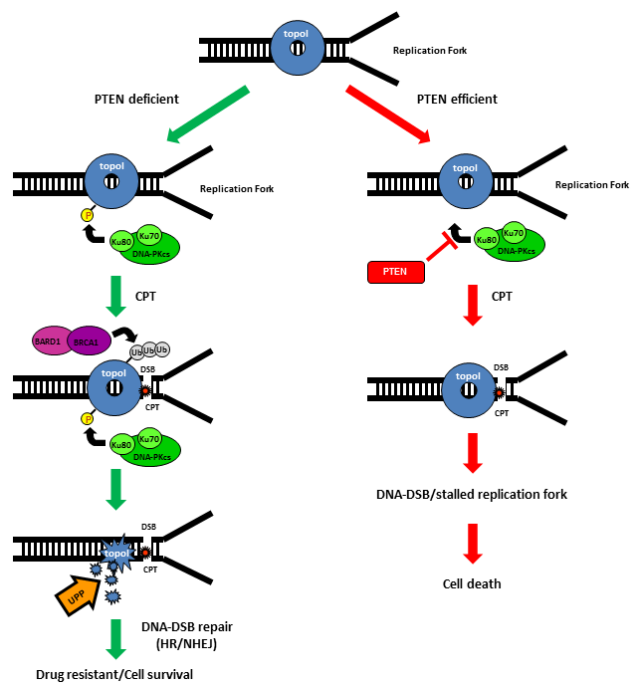
Schematic representation of UPP mediated degradation of topoI in response to CPT

In sum, CPT-induced proteasomal degradation of topoI determines CPT response; a higher basal level of DNA-PKcs dependent topoI-pS10 ensures rapid degradation and CPT resistance. BRCA1 E3 ligase function is critical in this pathway and PTEN is one of the upstream effector of this pathway (Figure 7).

## MATERIAL AND METHODS

### Tissue culture

#### Cell maintenance and growing

Mouse embryo fibroblasts (MEF) reconstituted for stable expression of DNA-PKcs (SCH8, DNA-PK +/+), wild type scid mice MEF (ScSV3, DNA-PK −/−) were kind gifts from Dr. Frederic Alt lab. HeLa cells, HCT-15 cells, BT-474 cells, HCC-1937 cells, MDA-MB-231 cells, SUM-52 cells, BT-549 cells, BT-20, HCT-116, Colo 205, H69, H249 and H526 were obtained from ATCC. MO59K cells and MO59J cells were kind gifts from Dr. Lees-Miller lab. SCH8 cells, ScSV3 cells, HeLa cells and MDA-MB-231 cells were grown and maintained in DMEM containing 10% fetal bovine serum, 2mM L-Glutamine, 100 units/ml of Streptomycin and 100 units/mL of Penicillin. HuMEC supplement (Invitrogen) was also used for SUM52 cells. BT474 cells, BT-549 cells, BT-20 cells, HCC-1937 cells, HCT-15 cells, HCT-116 cells, Colo 205 cells, H69 cells, H249 cells and H526 cells were maintained in RPMI containing 10% FBS and pen-strep. MO59K and MO59J cells were maintained in DMEM/F12 containing 10% FBS, 1mM MEM sodium pyruvate, 0.1mM NEAA and pen-strep. All cells were grown at 37°C and 5% CO_2_ in a humidified cell culture incubator.

#### Cell synchronization

MEF cells were phase-synchronized by serum starvation. Cells were incubated for 30 hours in DMEM containing 0.1% FBS followed by 16 hour incubation in complete medium containing 10% FBS.

#### Drug treatment

TopoI inhibitor treatment was performed using various concentrations of either irinotecan (Sigma-Aldrich) or SN-38 (Tocris). Cells were treated with 0.5 µM DNA-PK inhibitor NU7026 (EMD-CALBIOCHEM).

### Cell viability assay

In order to measure cell growth inhibition with the addition of CPTs, a Celigo (Cyntellect, Inc.) cell counting machine was used. Cells were incubated in a Hoechst 33258 dye for 15 minutes and between 5,000 and 10,000 cells per well were plated in a black 96-well plate (Corning), according to cell doubling time and size. An initial baseline cell count was taken 24 hours after plating and averaged over the entire 96 wells, giving a time zero count (Tz). Following the initial reading, the plate was divided into 16-wells each of control, 10 nM, 25 nM, 50 nM, 100 nM and 200 nM SN-38 treatments. The drug was replaced by fresh media after 24 hours of treatment. 72 hours following initial treatment of the cells, the plates were once again read using the Celigo fluorescent cell counting machine. Control growth (C) was then calculated by taking the average of the 16 control wells and subtracting by the overall plate Tz average. Test growth (Ti) was similarly averaged of 16 wells and subtracted from the plate Tz average. Overall percentage growth inhibition for each of the drug concentration levels was calculated using a formula adopted from the NCI *in vitro* cancer drug screen, where:

[(Ti-Tz)/(C-Tz)] x 100 for concentrations for which Ti ≥ Tz

[(Ti-Tz)/Tz] x 100 for concentrations for which Ti < Tz.

### Improvised GST pull down

An improvised GST pull down experiment was performed as described [21], and performed as follows:

#### GST fusion protein attached sepharose bead preparation

Purified GST (control) and GST-topoI were eluted from glutathione sepharose beads by elution buffer (10 mM reduced glutathione, 150mM NaCl, 50 mM Tris, pH 8.0). The eluted protein was dialyzed in PBS for twelve hours at 4°C. Protein concentration of dialyzed samples was determined by modified Bradford reagent (Bio-Rad). The eluted and dialyzed GST and GST-topoI protein was incubated with glutathione sepharose beads. 1.5 gm. of GST and GST-topoI were reattached to 1ml of GS beads. The reattached, PBS equilibrated GST and GST-topoI beads were poured into two empty columns, the columns were washed with PBS and used for pull down experiments.

#### Nuclear isolation

4×10^8^ cells were harvested. The cell pellet was re-suspended in buffer A (10 mM Hepes pH7.8, 10mM KCl, 0.1mM EDTA, 1mM DTT) with protease inhibitor cocktail (Roche). Cells were incubated for 10 minutes on ice and centrifuged for 5 minutes at 1500 g. The cells were resuspended in buffer A and homogenized (10-12 strokes) with type A hand held homogenizer. The homogenate was centrifuged at 1500g for five minutes, and the nuclear pallet was collected for protein extraction.

#### Nuclear extract

The nuclei was resuspended in buffer B (50 mM Hepes pH 7.8, 420mM KCl, 0.1mM EDTA, 5mM MgCl_2_, 1mM DTT and 2% glycerol with protease inhibitor cocktail). The resuspended nuclei were rotated for 30 minutes at 4°C and the nuclear extract was collected after centrifugation at 24,000g for 30 minutes. The nuclear extract was dialyzed in buffer C (30mM Tris pH 7.4, 5mM MgCl_2_, 1mM EDTA and 1mM DTT) for 20 hours with two buffer changes.

#### Protein complex isolation

An equal volume of the dialyzed nuclear extract was passed through GST and GST-topoI columns after equilibrating them with buffer C. The step was repeated three times to provide sufficient time for proteins in the extract to bind with GST and GST-topoI. The column was washed with twenty volume of buffer C. After the completion of the wash, 1 ml of buffer D (buffer C+150mM NaCl) was added to elute the interacting proteins. The eluted proteins were collected in five fractions of 200µl each. The proteins were further eluted and fractionated with 1 ml of buffer E (buffer C+500mM NaCl). The fractions containing topoI interacting proteins were analyzed by SDS-PAGE and silver staining.

### GST-topoI-deletion mutations

As described previously [53].

### BRCA1 silencing

#### Virus production

BRCA1 shRNA and control plasmids were purchased from Open Biosystems. All pLKO.1 plasmids were developed by the RNAi consortium (Boston, MA). Packaging and envelope plasmids were obtained from Addgene (Cambridge, MA). To produce virus, pLKO.1 plasmids, the packaging plasmid psPAX2, and the envelope plasmid pCMV-VSVg were simultaneously transfected into 293FT cells (Invitrogen). 18 hours after transfection, media was replaced with DMEM containing 30% FBS. Virus containing media was subsequently removed in 2 aliquots at 24 and 48 hours and frozen at -80°C.

#### Viral transduction

Virus transduction was completed by adding the appropriate amount of virus-containing media and 8µg/mL hexadimethrine bromide to growth media containing non-attached cells. Upon completion of transduction and attachment 24 hours later, virus-containing media was removed and replaced with fresh media containing 2.5 µg/mL puromycin. Transduced cells were selected for at least 2 days before use. Transduction efficiencies exceeded 95% in most cell types.

The following TRC designated plasmids were tested: TRCN0000039833, TRCN0000039834, TRCN0000039835, TRCN0000039836, and TRCN0000039837. It was determined that TRCN0000039834 resulted in the greatest knockdown.

### Immunoprecipitation and immunoblot analysis

As described previously [53].

### *In vitro* phosphorylation

Purified GST-topoI was incubated with DNA-PK in kinase buffer (20 mM Tris-HCl, pH 7.4, 10 mM MgCl2 and 10 mM MnCl2) containing [γ32P]ATP or cold ATP for 30 min at 30°C. The reaction products were analyzed by SDS-PAGE and autoradiography. Selected reaction products were also used for *in vitro* ubiquitination experiments.

### Protein identification by mass spectrometry

Proteins from coomassie/silver stained gels were excised and in gel digested as previously described [54]. Trypsin digested proteins were analyzed by mass spectrometry.

### Phosphopeptide enrichment for mass spectrometry

Following tryptic digestion of the protein, immobilized metal affinity chromatography (IMAC) was performed to enrich phosphorylated peptides as previously described [55]. Briefly, tryptic peptides were loaded onto a self-packed IMAC column (in fused silica capillary, 100 µm I.D., 10cm length) charged with Fe3+. Non-phosphorylated peptides were removed by rinsing with 20 µL of 25% acetonitrile in 100 mM NaCl. Peptides retained on the IMAC column were eluted with 250 mM sodium phosphate (pH 8.0) and loaded onto a reverse-phase C18 pre-column (100 µm I.D. x 10 cm length), which was then rinsed with 10 µL of 0.2 M acetic acid to remove phosphate buffer. After rinsing, the pre-column was attached to a reverse-phase C18 resolving column (50 µm I.D., 10 cm length) with an integrated electrospray tip. Peptides were then eluted from the column and analyzed by electrospray ionization liquid chromatography tandem MS on a QqTof mass spectrometer (QSTAR Elite, Applied Biosystems). MS/MS spectra of the five most intense peaks with 2 - 5 charge states in the full MS scan were acquired in information-dependent acquisition.

### Phosphopeptide sequencing and quantification

MS/MS spectra were extracted and searched using MASCOT (Matrix Science) against the human non-redundant protein database with trypsin specificity, two missed cleavages, precursor mass tolerance of 2.2 amu for the precursor ion and 0.15 for the fragment ion tolerance. Phosphorylation sites and peptide sequence assignments were validated and quantified by manual confirmation of raw MS/MS data.

### *In vitro* ubiquitination

Reactions on 700 ng of topo I were carried out in 10 mM HEPES (pH 7.9), 0.5 mM EDTA, 5 mM MgCl2, 2 mM NaF, 2 mM ATP, 60 mM KCl, 1 µM ubiquitin, 200 nM E1-His, 5 µM UbcH5c-His (E2) and 200 to 400 ng of BRCA1-Flag/BARD1 (E3). Negative controls were also established in the absence of BRCA1-Flag/BARD1. Reaction products were analyzed by SDS-PAGE and autoradiography.

### Development of HCC-1937 BRCA1 stable cell line

HCC-1937 cells with a known mutation in the *BRCA1* gene yielding a truncated and non-functional BRCA1 protein were transduced with lentivirus containing the full-length wild type (WT) BRCA1. Following infection, the cells were selected for blasticidin resistance. Two colonies were finally selected and BRCA1 expression was confirmed by immunoblot with anti-BRCA1 antibody (Santa Cruz, CA) (data not shown).

### TopoI ubiquitination in cells

Following 30 minutes of MG-132 (5 μM) treatment, cells were treated with 1 μM SN-38 for 30 minutes. Cells were lysed according to the procedure described [16]. Briefly, cells were lysed in 0.2 N NaOH with 2 mM EDTA. Cell lysates were then sonicated and neutralized with 2N HCl, followed by the addition of 10% Nonidet P-40, 1M Tris, pH 7.4, 0.1 M MgCl_2_, 0.1M CaCl_2_, 10 mM dithiothreitol, 1 mM EGTA, and 100 μg/ml of each leupeptin, pepstatin, and aprotinin. Micrococcal nuclease (10 μg/ml) was added and incubated for 30 minutes at room temperature. Reactions were stopped by the addition of 0.1 M EGTA. Micrococal nuclease treated cell lysates were subjected to immunoprecipitation using scleroderma patient serum (containing high anti-topoI titer). Protein A bound magnetic nanoparticles (Miltenyi) were then added to the lysate and further rotated for 1 hour at 4°C. Immunoprecipitates were washed with low salt buffer using a magnetic column (Miltenyi). Immunoprecipitates were eluted from the column and analyzed by immunoblot analysis with pan or lysine-specific (K-48 or K-63) anti-ubiquitin antibodies (Cell Signaling, MA), as well as anti-topoI (BD Biosciences).

### Developing topoI-pS10 phosphospecific monoclonal antibody

Mouse monoclonal antibody against topoI-pS10 was produced and purified at the hybridoma core facility of Dana-Farber Cancer Institute, Boston. A phosphopeptide containing the N-terminus of topoI with phosphoserine 10 was used to raise the antibody. Several clones were screened by ELISA assays and 10 clones were selected and screened for IHC. After the screen, clone 357.3.1C1.H5.H7 was finally selected for large volume culture and purification of the antibody.

### Integrating EGFP following the *hTOP1* gene in HCT-15 cells

Summary sequences of plasmids used in this study can be found in Supplementary Table 1. Roughly 1000 bases 5’ and 3’ of the genomic sequence flanking the last *hTOP1* exon in HCT-15 cells were sequenced to identify and account for any cell-type specific polymorphisms. To do so, multiple PCR products spanning the genomic sequence were amplified using Phusion DNA Polymerase (New England Biolabs). The resulting PCR amplicons were cloned using the Zero Blunt TOPO PCR Cloning Kit (Invitrogen), transformed into E.coli XL-1 blue competent cells, and ~20-25 colonies were grown overnight at 37°C in TB media prior to miniprep and sanger sequencing (MGH DNA Core). A single guide-RNA (sgRNA) targeting the *hTOP1* stop codon was designed so that the SpCas9 binding site would be destroyed following gene conversion. To generate the sgRNA plasmid, oligonucleotides corresponding to the spacer sequence of the target site were annealed and ligated into BsmBI cut BPK1520 [56]. The homologous recombination donor plasmid designed to create the topoI-EGFP fusion protein was generated by Gibson assembly into the NheI and HindIII sites of pUC19. Regions of the genomic sequence 5’ and 3’ of the *hTOP1* stop codon were assembled with an EGFP-P2A-*Pac* (for puromycin resistance) fusion cassette to generate the final donor plasmid (with 5’ and 3’ homology regions of 934 and 824 bases, respectively). Transfections into HCT-15 cells were performed by lipofectamine(Thermo Fisher Sceintific) with: 1) 2µg of a wild-type *Streptococcus pyogenes* Cas9 expression plasmid (MSP469) [56], 2) 1µg of the sgRNA expression plasmid (MMW134) and 3) 2µg of the homologous recombination donor plasmid (MMW274). After 7 days of the transfection, cells were selected with 4µg/ml puromycin. After 14 days of selection, the cells were sorted by MoFlo Legacy (Beckman Coulter). The sorted cells were maintained in 2µg/ml puromycin contained media. Precise incorporation of the genome editing cassette in single cell cloned HCT-15 *hTOP1*-EGFP cells was confirmed by PCR amplifying the genomic locus and sequencing. Also, western blot analysis and immunofluorescence was done to characterize the genome edited cells (Figure 4D and 4E).

### Quantifying EGFP intensity by plate reader

Genome edited topoI-EGFP HCT-15 cells were grown in 96 well plate black clear bottoms. Cells were treated with 2.5µM SN38 for indicated time points. Then the media was changed to PBS and EGFP intensity was measured by plate reader (Tecan).

### DNA-PKcs kinase assay

0.05µg of DNA-PKcs and 0.03µg of Ku70/80 were incubated with double-strand DNA in kinase buffer (50mM HEPES pH 7.5, 10mM MgCl2, 75mM KCl and 0.2mM ATP) for 30mins at 30°C. Then DNA-PK inhibitor; NU7026 was added and incubated for 5mins at room temperature. Then PTEN was added and incubated for 30mins at 30°C. The proteins were boiled in an SDS sample buffer and separated by SDS-PAGE (5% polyacrylamide) and analyzed by immunoblotting with anti-phospho DNA-PK S2056 antibody.

### Immunofluorescence

Cells were grown on a sterilized coverslip. After the treatment, the coverslip was washed twice with ice cold PBS, fixed with 3.7% formaldehyde, and permeabilized with 0.2% Triton X-100. After blocking with 3% BSA for 60 minutes, the coverslip was incubated with monoclonal anti-phospho DNAPK S2056 (abcam) antibody, monoclonal anti-topoI antibody (BD) or monoclonal anti-phospho topoI S10 antibody followed by Alexa fluor 488-conjugated goat anti-rabbit IgG (Molecular Probes) antibody or Alexa fluor 594-conjugated goat anti-mouse IgG (Molecular Probes). The cell nuclei were stained with DAPI. Fluorescent imaging was performed utilizing Leica SP5 fluorescence microscopy driven by LAS software (Leica Microsystems).

### Flow cytometry

After the addition of drugs at indicated time points, cells were collected and washed with ice cold PBS and fixed with ice cold 70% ethanol. Cells were treated with 500 µg/ml of RNase A (Sigma) and subsequently stained with 50 µg/ml of propidium iodide (PI, Sigma) for 15 minutes at 37°C. The populations of cells stained by PI were then analysed by FACS calibre flow cytometer (Beckton Dickinson).

### Clonogenic assay

C2 and PTEN shRNA ells were treated with variable concentrations of SN38 (notably 0uM, 0.5nM, 1nM, 2.5nM, 5nM, 7.5nM) for 24 hrs. Then cells were plated as 50 cells per well in 6 well plate in normal media. After 12 days when colonies were apparent, cells were fixed with 6% glutaraldehyde and 0.5% crystal violet for 15 min. Then cells were washed in a tub of water. The colonies per well were counted.

### Statistical analysis

In the siRNA library screening, we calculated the Z-score using the formula (mean gene-mean control)/sd (control). Bonferonni corrections were used to determine the significance threshold (*p* = 0.0025). For other statistical analysis, a student *t*-test with one-sided analysis was used.

## SUPPLEMENTARY MATERIALS FIGURES AND TABLE




