# Supplementary material for: Camptothecin resistance is determined by the regulation of topoisomerase I degradation mediated by ubiquitin proteasome pathway
Source: Oncotarget. 2017 Mar 18;8(27):43733–51. doi: 10.18632/oncotarget.16376 (PMC5546437; doi:10.18632/oncotarget.16376)
Supplement: Supplementary file 1 [file oncotarget-08-43733-s001.pdf]

Camptothecin resistance is determined by the regulation of topoisomerase I degradation mediated by ubiquitin proteasome pathway

Supplementary Material

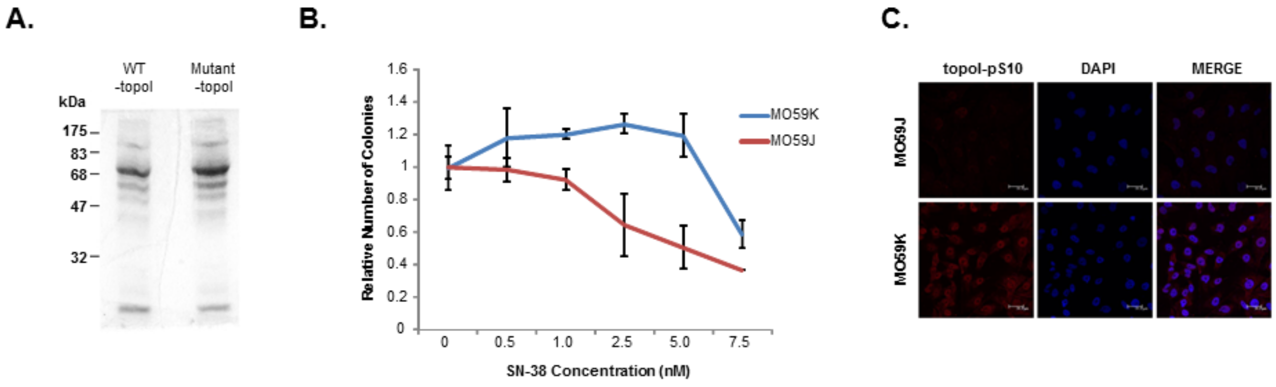

Supplementary Figure S1. Related to Figure 1 and 2
A. Loading control in wild type topoI and mutant topoI related to Figure 1F. **B.** Clonogenic assay in MO59K and MO59J cells. MO59K cell has normal level of DNA-PKcs and MO59J cell is DNA-PKcs deficient cells. Cells were treated with indicated concentration of SN-38 for 24 hours. **C.** TopoI-pS10 level in MO59J and MO59K cells.

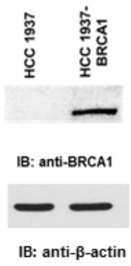

Figure S2. Related to Figure 3
Establishment of stably expressed BRCA1 in HCC1937 cell BRCA1-WT was stably expressed in HCC1937 (BRCA1 deletion mutant cells), referred to as HCC1937-BRCA1. HCC1937 and HCC1937-BRCA1 cells were lysed and cell lysates were analyzed by immunoblot with anti-BRCA1 and anti-β-actin.

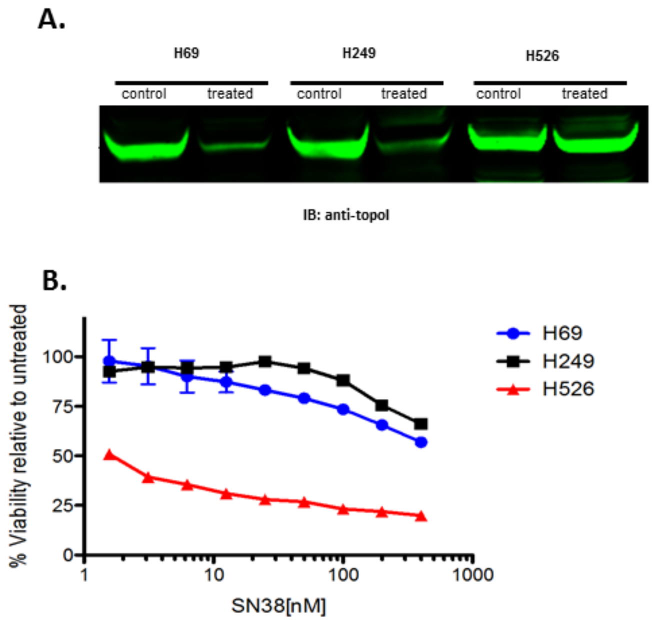

Figure S3. Related to Figure 4
C. SCLC cell lines H69, H249, and H526 were treated with 1 μM SN-38 for 3 hours, and cell lysates from control and treated cells were immunoblot with anti-topoI antibody. **D,** Cell survival in the presence of various concentrations of SN-38 was determined by MTT after 72 hours treatment.

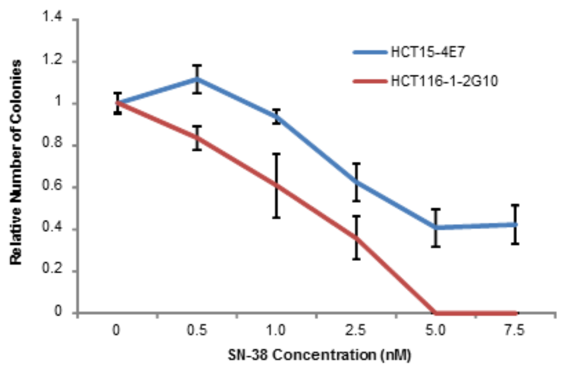

Figure S4. Related to Figure 5
Clonogenic assay in genetically engineered HCT15-4E7 cells and HCT116-1-2G10 cells. HCT15 is a resistant cell line to CPT and HCT116 is a sensitive cell line to CPT. Cells were treated with various SN-38 as indicated for 24hours.

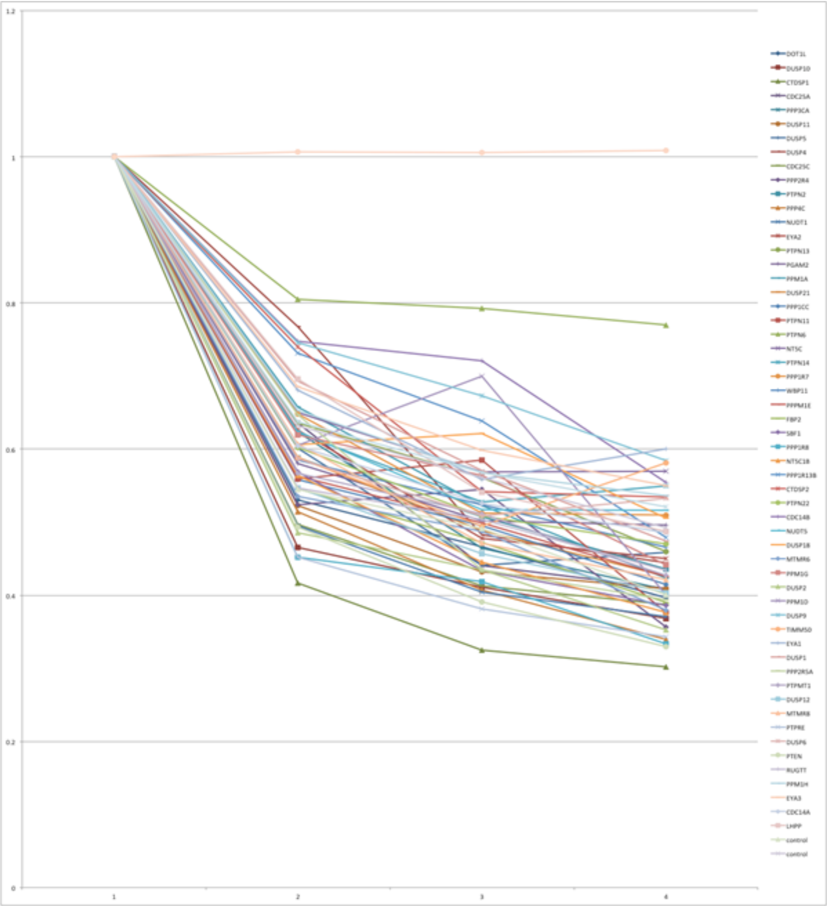

Figure S5. Related to Figure 6
siRNA library screening for 56 nuclear phosphatases.
56 nuclear phosphatases were silenced using siRNA library in HCT15-4E7 cells. Then the cells were treated with 2.5mM SN-38. After certain time points (2, 4, 6hours), the GFP fluorescence intensity was quantitatively analyzed by plate reader.

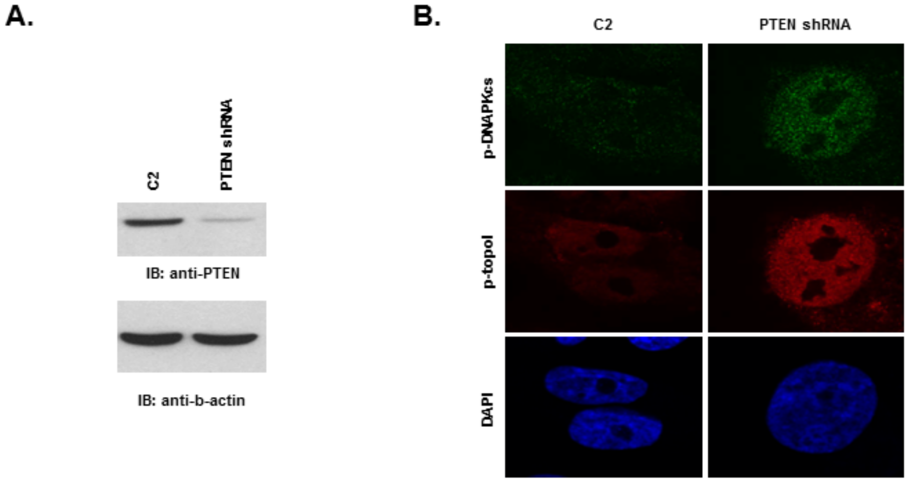

Figure S6. Related to Figure 6

A. Stably PTEN silenced MDA-MB231 (PTEN shRNA) and control cells with WT-PTEN (C2) were lyzed and the cell lysates were analyzed by immunoblot analysis with anti-PTEN and anti-β-actin antibodies. **B.** C2 and PTEN-/- cells were fixed in 3.7% formaldehyde. Followed by cell permeabilization the cells were incubated with anti-p-DNA-PKcs and anti-p-TopoI antibody (1:100). Then incubated with 0.1%DAPI. Cells were visualized by Leica S5 confocal microscope.

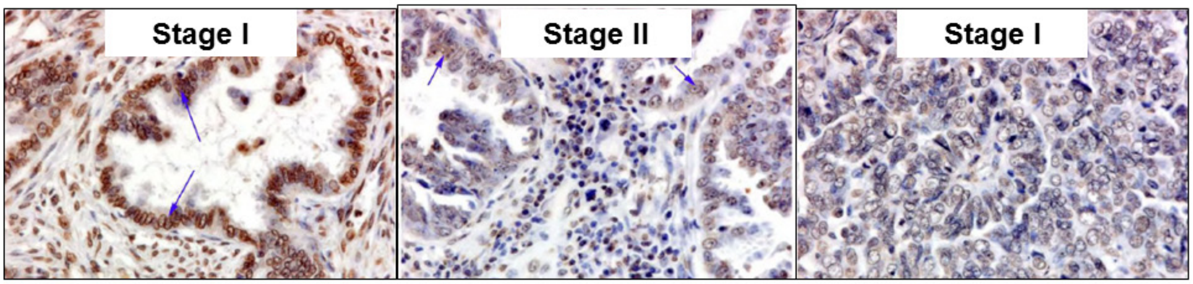

Figure S7:

Ovarian carcinoma tissue micro-array obtained from USBiomax (BC111109) was stained with anti-topal-pS10. Representative tissue: two stage I carcinoma and one stage II carcinoma, are shown. Blue arrows indicate nuclear labelling in cancer cells.
