# Supplementary material for: Camptothecin resistance is determined by the regulation of topoisomerase I degradation mediated by ubiquitin proteasome pathway
Source: Oncotarget. 2017 Mar 18;8(27):43733–51. doi: 10.18632/oncotarget.16376 (PMC5546437; doi:10.18632/oncotarget.16376)
Supplement: Supplementary file 2 [file oncotarget-08-43733-s002.docx]

**Supplementary Table 1: Sequences of plasmids used for genome editing:**

MSP469: CMV-T7-humanSpCas9-VQR(D1135V, R1335Q, T1337R)-NLS-3xFLAG (Addgene #65771)

Human codon optimized *S. pyogenes* Cas9 colored in blue, modified codons in red, NLS underlined, 3xFLAG tag in **bold**

ATGGATAAAAAGTATTCTATTGGTTTAGACATCGGCACTAATTCCGTTGGATGGGCTGTCATAACCGATGAATACAAAGTACCTTCAAAGAAATTTAAGGTGTTGGGGAACACAGACCGTCATTCGATTAAAAAGAATCTTATCGGTGCCCTCCTATTCGATAGTGGCGAAACGGCAGAGGCGACTCGCCTGAAACGAACCGCTCGGAGAAGGTATACACGTCGCAAGAACCGAATATGTTACTTACAAGAAATTTTTAGCAATGAGATGGCCAAAGTTGACGATTCTTTCTTTCACCGTTTGGAAGAGTCCTTCCTTGTCGAAGAGGACAAGAAACATGAACGGCACCCCATCTTTGGAAACATAGTAGATGAGGTGGCATATCATGAAAAGTACCCAACGATTTATCACCTCAGAAAAAAGCTAGTTGACTCAACTGATAAAGCGGACCTGAGGTTAATCTACTTGGCTCTTGCCCATATGATAAAGTTCCGTGGGCACTTTCTCATTGAGGGTGATCTAAATCCGGACAACTCGGATGTCGACAAACTGTTCATCCAGTTAGTACAAACCTATAATCAGTTGTTTGAAGAGAACCCTATAAATGCAAGTGGCGTGGATGCGAAGGCTATTCTTAGCGCCCGCCTCTCTAAATCCCGACGGCTAGAAAACCTGATCGCACAATTACCCGGAGAGAAGAAAAATGGGTTGTTCGGTAACCTTATAGCGCTCTCACTAGGCCTGACACCAAATTTTAAGTCGAACTTCGACTTAGCTGAAGATGCCAAATTGCAGCTTAGTAAGGACACGTACGATGACGATCTCGACAATCTACTGGCACAAATTGGAGATCAGTATGCGGACTTATTTTTGGCTGCCAAAAACCTTAGCGATGCAATCCTCCTATCTGACATACTGAGAGTTAATACTGAGATTACCAAGGCGCCGTTATCCGCTTCAATGATCAAAAGGTACGATGAACATCACCAAGACTTGACACTTCTCAAGGCCCTAGTCCGTCAGCAACTGCCTGAGAAATATAAGGAAATATTCTTTGATCAGTCGAAAAACGGGTACGCAGGTTATATTGACGGCGGAGCGAGTCAAGAGGAATTCTACAAGTTTATCAAACCCATATTAGAGAAGATGGATGGGACGGAAGAGTTGCTTGTAAAACTCAATCGCGAAGATCTACTGCGAAAGCAGCGGACTTTCGACAACGGTAGCATTCCACATCAAATCCACTTAGGCGAATTGCATGCTATACTTAGAAGGCAGGAGGATTTTTATCCGTTCCTCAAAGACAATCGTGAAAAGATTGAGAAAATCCTAACCTTTCGCATACCTTACTATGTGGGACCCCTGGCCCGAGGGAACTCTCGGTTCGCATGGATGACAAGAAAGTCCGAAGAAACGATTACTCCATGGAATTTTGAGGAAGTTGTCGATAAAGGTGCGTCAGCTCAATCGTTCATCGAGAGGATGACCAACTTTGACAAGAATTTACCGAACGAAAAAGTATTGCCTAAGCACAGTTTACTTTACGAGTATTTCACAGTGTACAATGAACTCACGAAAGTTAAGTATGTCACTGAGGGCATGCGTAAACCCGCCTTTCTAAGCGGAGAACAGAAGAAAGCAATAGTAGATCTGTTATTCAAGACCAACCGCAAAGTGACAGTTAAGCAATTGAAAGAGGACTACTTTAAGAAAATTGAATGCTTCGATTCTGTCGAGATCTCCGGGGTAGAAGATCGATTTAATGCGTCACTTGGTACGTATCATGACCTCCTAAAGATAATTAAAGATAAGGACTTCCTGGATAACGAAGAGAATGAAGATATCTTAGAAGATATAGTGTTGACTCTTACCCTCTTTGAAGATCGGGAAATGATTGAGGAAAGACTAAAAACATACGCTCACCTGTTCGACGATAAGGTTATGAAACAGTTAAAGAGGCGTCGCTATACGGGCTGGGGACGATTGTCGCGGAAACTTATCAACGGGATAAGAGACAAGCAAAGTGGTAAAACTATTCTCGATTTTCTAAAGAGCGACGGCTTCGCCAATAGGAACTTTATGCAGCTGATCCATGATGACTCTTTAACCTTCAAAGAGGATATACAAAAGGCACAGGTTTCCGGACAAGGGGACTCATTGCACGAACATATTGCGAATCTTGCTGGTTCGCCAGCCATCAAAAAGGGCATACTCCAGACAGTCAAAGTAGTGGATGAGCTAGTTAAGGTCATGGGACGTCACAAACCGGAAAACATTGTAATCGAGATGGCACGCGAAAATCAAACGACTCAGAAGGGGCAAAAAAACAGTCGAGAGCGGATGAAGAGAATAGAAGAGGGTATTAAAGAACTGGGCAGCCAGATCTTAAAGGAGCATCCTGTGGAAAATACCCAATTGCAGAACGAGAAACTTTACCTCTATTACCTACAAAATGGAAGGGACATGTATGTTGATCAGGAACTGGACATAAACCGTTTATCTGATTACGACGTCGATCACATTGTACCCCAATCCTTTTTGAAGGACGATTCAATCGACAATAAAGTGCTTACACGCTCGGATAAGAACCGAGGGAAAAGTGACAATGTTCCAAGCGAGGAAGTCGTAAAGAAAATGAAGAACTATTGGCGGCAGCTCCTAAATGCGAAACTGATAACGCAAAGAAAGTTCGATAACTTAACTAAAGCTGAGAGGGGTGGCTTGTCTGAACTTGACAAGGCCGGATTTATTAAACGTCAGCTCGTGGAAACCCGCCAAATCACAAAGCATGTTGCACAGATACTAGATTCCCGAATGAATACGAAATACGACGAGAACGATAAGCTGATTCGGGAAGTCAAAGTAATCACTTTAAAGTCAAAATTGGTGTCGGACTTCAGAAAGGATTTTCAATTCTATAAAGTTAGGGAGATAAATAACTACCACCATGCGCACGACGCTTATCTTAATGCCGTCGTAGGGACCGCACTCATTAAGAAATACCCGAAGCTAGAAAGTGAGTTTGTGTATGGTGATTACAAAGTTTATGACGTCCGTAAGATGATCGCGAAAAGCGAACAGGAGATAGGCAAGGCTACAGCCAAATACTTCTTTTATTCTAACATTATGAATTTCTTTAAGACGGAAATCACTCTGGCAAACGGAGAGATACGCAAACGACCTTTAATTGAAACCAATGGGGAGACAGGTGAAATCGTATGGGATAAGGGCCGGGACTTCGCGACGGTGAGAAAAGTTTTGTCCATGCCCCAAGTCAACATAGTAAAGAAAACTGAGGTGCAGACCGGAGGGTTTTCAAAGGAATCGATTCTTCCAAAAAGGAATAGTGATAAGCTCATCGCTCGTAAAAAGGACTGGGACCCGAAAAAGTACGGTGGCTTCGTGAGCCCTACAGTTGCCTATTCTGTCCTAGTAGTGGCAAAAGTTGAGAAGGGAAAATCCAAGAAACTGAAGTCAGTCAAAGAATTATTGGGGATAACGATTATGGAGCGCTCGTCTTTTGAAAAGAACCCCATCGACTTCCTTGAGGCGAAAGGTTACAAGGAAGTAAAAAAGGATCTCATAATTAAACTACCAAAGTATAGTCTGTTTGAGTTAGAAAATGGCCGAAAACGGATGTTGGCTAGCGCCGGAGAGCTTCAAAAGGGGAACGAACTCGCACTACCGTCTAAATACGTGAATTTCCTGTATTTAGCGTCCCATTACGAGAAGTTGAAAGGTTCACCTGAAGATAACGAACAGAAGCAACTTTTTGTTGAGCAGCACAAACATTATCTCGACGAAATCATAGAGCAAATTTCGGAATTCAGTAAGAGAGTCATCCTAGCTGATGCCAATCTGGACAAAGTATTAAGCGCATACAACAAGCACAGGGATAAACCCATACGTGAGCAGGCGGAAAATATTATCCATTTGTTTACTCTTACCAACCTCGGCGCTCCAGCCGCATTCAAGTATTTTGACACAACGATAGATCGCAAACAGTACAGATCTACCAAGGAGGTGCTAGACGCGACACTGATTCACCAATCCATCACGGGATTATATGAAACTCGGATAGATTTGTCACAGCTTGGGGGTGACGGATCCCCCAAGAAGAAGAGGAAAGTCTCGAGC**GACTACAAAGACCATGACGGTGATTATAAAGATCATGACATCGATTACAAGGATGACGATGACAAG**TGA

BPK1520: U6-BsmBIcassette-Sp-sgRNA (Addgene #65777)

U6 promoter in green, BsmBI sites underlined, *S. pyogenes* sgRNA colored in purple, U6 terminator underlined

TGTACAAAAAAGCAGGCTTTAAAGGAACCAATTCAGTCGACTGGATCCGGTACCAAGGTCGGGCAGGAAGAGGGCCTATTTCCCATGATTCCTTCATATTTGCATATACGATACAAGGCTGTTAGAGAGATAATTAGAATTAATTTGACTGTAAACACAAAGATATTAGTACAAAATACGTGACGTAGAAAGTAATAATTTCTTGGGTAGTTTGCAGTTTTAAAATTATGTTTTAAAATGGACTATCATATGCTTACCGTAACTTGAAAGTATTTCGATTTCTTGGCTTTATATATCTTGTGGAAAGGACGAAACACCGGAGACGATTAATGCGTCTCCGTTTTAGAGCTAGAAATAGCAAGTTAAAATAAGGCTAGTCCGTTATCAACTTGAAAAAGTGGCACCGAGTCGGTGCTTTTTTT

MMW134: U6-SpCas9(VQR variant)-sgRNA targeted to stop codon of *topo1* (NGA PAM site)

U6 promoter in green, target spacer underlined, *S. pyogenes* sgRNA colored in purple, U6 terminator underlined

TGTACAAAAAAGCAGGCTTTAAAGGAACCAATTCAGTCGACTGGATCCGGTACCAAGGTCGGGCAGGAAGAGGGCCTATTTCCCATGATTCCTTCATATTTGCATATACGATACAAGGCTGTTAGAGAGATAATTAGAATTAATTTGACTGTAAACACAAAGATATTAGTACAAAATACGTGACGTAGAAAGTAATAATTTCTTGGGTAGTTTGCAGTTTTAAAATTATGTTTTAAAATGGACTATCATATGCTTACCGTAACTTGAAAGTATTTCGATTTCTTGGCTTTATATATCTTGTGGAAAGGACGAAACACCATGAGTTTTAGCCAGTCTCAGTTTTAGAGCTAGAAATAGCAAGTTAAAATAAGGCTAGTCCGTTATCAACTTGAAAAAGTGGCACCGAGTCGGTGCTTTTTTT

MMW274: *topo1*-EGFP-P2A-pac HR donor plasmid

5’ homology region in blue, final exon of top1 in yellow, linker sequence underlined, EGFP in green, P2A in orange, puromycin N-acetyl-transferase gene in grey, stop codon in red, and 3’ homology region in purple. GGTCAGTGCTGTTACACTGCCTTGTAGTGTATATTTTTAAGAGGCAGGTGCCATCCCTATTCCTAATGGAACTTTAGGACAGGAATGGAAACTCTTAGCTTCTGGAAGAATAGGAAAGAGCCCATCCATCTCTACACTTCAACAAAACTTTTCGTTGAATTTTTTTGCAGTTGAAGGTAGGAACAAGAGATAAAGATGAGAATACAGATCTGAGCAACTTACACAGAGAGGCAGAGGGACCTTTAACAAAAGCAGATAAATACTAGGTTTCTCTGTGTATCACACACAGTGTCACATAACATCTGAAACAAGTGGCTTTGTTATGGAAGATGTTTAGTTTGAGCTGTTAAGTTCTGAGCATAGGTGGAGATATCCTCCCCTATGGCACTTGCTAGTCCGGGCTAAAGTTTCCATCTAGGTCTTTTGTACCTCTTTCTGCTCGTTTTGCCTTGTTTGGTGCTAGAGATTTGGTTAGCTCTTAAAAGGCAATATAGTATAGTGGTTAAGAACATGGAGAAAAACTGCTTGGGTTCAAATTTCAGGTCACTTACCAGCTAGCTGTGTGAGAAAATCAGTAAATTACCTAACGTCTCTGCCTCAGTTTCATCTGTACGTTTTTCACTTGTACATCTGCAGAAGCACCTACTTTAGAGATCACTGCCAAGATTAAATGAGTTGATCACATAAAACCTTTAGAACATGCTTGGTGCACTATTAATTTGCATCCTCACTAGACAGATGTGAAAGAGAAGATGGAACATCTGACCCTGGGCCTCAGATATGGGCCATTGCTGAGTCACCCTAATCCCCCCCTTATTTCTCCTTTGTTTGCAGGTGCAAGAAGTGGGGTGTCCCAATTGAGAAGATTTACAACAAAACCCAGCGGGAGAAGTTTGCCTGGGCCATTGACATGGCTGATGAAGACTATGAGTTTGCAGGAGGCGGTGGAAGCGGGATGGTGAGCAAGGGCGAGGAGCTGTTCACCGGGGTGGTGCCCATCCTGGTCGAGCTGGACGGCGACGTAAACGGCCACAAGTTCAGCGTGTCCGGCGAGGGCGAGGGCGATGCCACCTACGGCAAGCTGACCCTGAAGTTCATCTGCACCACCGGCAAGCTGCCCGTGCCCTGGCCCACCCTCGTGACCACCCTGACCTACGGCGTGCAGTGCTTCAGCCGCTACCCCGACCACATGAAGCAGCACGACTTCTTCAAGTCCGCCATGCCCGAAGGCTACGTCCAGGAGCGCACCATCTTCTTCAAGGACGACGGCAACTACAAGACCCGCGCCGAGGTGAAGTTCGAGGGCGACACCCTGGTGAACCGCATCGAGCTGAAGGGCATCGACTTCAAGGAGGACGGCAACATCCTGGGGCACAAGCTGGAGTACAACTACAACAGCCACAACGTCTATATCATGGCCGACAAGCAGAAGAACGGCATCAAGGTGAACTTCAAGATCCGCCACAACATCGAGGACGGCAGCGTGCAGCTCGCCGACCACTACCAGCAGAACACCCCCATCGGCGACGGCCCCGTGCTGCTGCCCGACAACCACTACCTGAGCACCCAGTCCGCCCTGAGCAAAGACCCCAACGAGAAGCGCGATCACATGGTCCTGCTGGAGTTCGTGACCGCCGCCGGGATCACTCTCGGCATGGACGAGCTGTACAAGGGATCCGGCGCAACAAACTTCTCTCTGCTGAAACAAGCCGGAGATGTCGAAGAGAATCCTGGACCGACCGAGTACAAGCCCACGGTGCGCCTCGCCACCCGCGACGACGTCCCCAGGGCCGTACGCACCCTCGCCGCCGCGTTCGCCGACTACCCCGCCACGCGCCACACCGTCGATCCGGACCGCCACATCGAGCGGGTCACCGAGCTGCAAGAACTCTTCCTCACGCGCGTCGGGCTCGACATCGGCAAGGTGTGGGTCGCGGACGACGGCGCCGCGGTGGCGGTCTGGACCACGCCGGAGAGCGTCGAAGCGGGGGCGGTGTTCGCCGAGATCGGCCCGCGCATGGCCGAGTTGAGCGGTTCCCGGCTGGCCGCGCAGCAACAGATGGAAGGCCTCCTGGCGCCGCACCGGCCCAAGGAGCCCGCGTGGTTCCTGGCCACCGTCGGAGTCTCGCCCGACCACCAGGGCAAGGGTCTGGGCAGCGCCGTCGTGCTCCCCGGAGTGGAGGCGGCCGAGCGCGCCGGGGTGCCCGCCTTCCTGGAGACCTCCGCGCCCCGCAACCTCCCCTTCTACGAGCGGCTCGGCTTCACCGTCACCGCCGACGTCGAGGTGCCCGAAGGACCGCGCACCTGGTGCATGACCCGCAAGCCCGGTGCCTAGCCAGTCTCAAGAGGCAGGGTTCTGTGAAAAGGAACAGTGTGGTTTGGGAGAAATGGATAAACTGAGCCTCGCTTGCCCTCGTGCCTGGGGGAGAGAGGCAACAAGTCTTAACAAACCAACATCTTTGAGAAAAGATAAACCTGGAGATAATAACATAAGGGAGAGCTGAGCCAGTTGTCCTGTGGACAACTTATTTAAAAATATTTCAGATATCAAAATTCTAGCTGTATGATTTGTTTTGAATTTTGTTTTTATTTTCAAGAGGGCAAATGGATGGGAATTTGTCAGCGTTCTACCAGGCAAATTCACTGTTTCACTGAAATGTTTGGATTCTCTTAGCTACTGTATGAAAAGTCCGATTATATTGGTGTGTTCTTACAGTTGGGGTTTTGCAATAACTTCTGTATTTTAATATAAATAAATTCCTAAACTCCCTCCCCTCTCCCCCATTTCAGGAATTTAAAATTAAGTAGAACAAAAAAACCCAGCGCACCTGTTAGAGTCGTCACTCTCTATTGTCATGGGGATCAATTTTCATTAAACTTGAAGCAGTCGTGGCTTTGGCAGTGTTTTGGTTCAGACACCTGTTCACAGAAAAAGCATGATGGGAAAATATTTCCTGACTTGAGTGTTCCTTTTTAAATGTGAATTTTTATTTCTTTTTAATTATTTTAAAATATTTAAACCTTTTTCTTGATCTTACAGATCGTGTAGATTGGGGTTGGGGAAGGATGAAGGGCGAGTGAGTCTATGGATAATGAAATAATCAGTGACTGAAACCATTTTCCCATCATCCTTTGTTCTGAGCATTCGCTGTACCC
